# The tale of two Ions Na^+^ and Cl^−^: unraveling onion plant responses to varying salt treatments

**DOI:** 10.1186/s12870-024-05719-9

**Published:** 2024-10-29

**Authors:** M. L. Romo-Pérez, C. H. Weinert, B. Egert, S. E. Kulling, C. Zörb

**Affiliations:** 1 University of Hohenheim, Institute of Crop Science, Quality of Plant Products 340e, Schloss Westflügel, Stuttgart, 70599 Germany; 2 Department of Safety and Quality of Fruit and Vegetables, Max Rubner-Institut, Haid-und-Neu-Straße 9, Karlsruhe, 76131 Germany

**Keywords:** Salinity, Allium Cepa L., Sodium (Na^+^), Chloride (Cl^−^), Organic Acids, Tricarboxylic Acid Cycle (TCA), Metabolomics

## Abstract

**Background:**

Exploring the adaptive responses of onions (*Allium cepa* L.) to salinity reveals a critical challenge for this salt-sensitive crop. While previous studies have concentrated on the effects of sodium (Na^+^), this research highlights the substantial yet less-explored impact of chloride (Cl^−^) accumulation. Two onion varieties were subjected to treatments with different sodium and chloride containing salts to observe early metabolic responses without causing toxicity.

**Results:**

The initial effects of salinity on onions showed increased concentrations of both ions, with Cl^−^ having a more pronounced impact on metabolic profiles than Na^+^. Onions initially adapt to salinity by first altering their organic acid concentrations, which are critical for essential functions such as energy production and stress response. The landrace Birnförmige exhibited more effective regulation of its Na^+^/K^+^ balance and a milder response to Cl^−^ compared to the hybrid Hytech. Metabolic alterations were analyzed using advanced techniques, revealing specific responses in leaves and bulbs to Cl^−^ accumulation, with significant changes observed in organic acids involved in the TCA cycle, such as fumaric acid, and succinic acid, in both varieties. Additionally, there was a variety-specific increase in ethanolamine in Birnförmige and lysine in Hytech in response to Cl^−^ accumulation.

**Conclusion:**

This comprehensive study offers new insights into onion ion regulation and stress adaptation during the initial stages of salinity exposure, emphasizing the importance of considering both Na^+^ and Cl^−^ when assessing plant responses to salinity.

**Supplementary Information:**

The online version contains supplementary material available at 10.1186/s12870-024-05719-9.

## Introduction

Onions rank as the second most widely cultivated vegetable globally, with production reaching an impressive 110 billion tons in 2022 [1]. Cultivating onions can be challenging in certain regions especially in irrigated lands, largely due to soil characteristics such as salinity [2]. Soil salinization remains a significant hurdle in agriculture, yet the adaptive mechanisms of vegetables like onions to such conditions are poorly understood. The repercussions of ions like Na^+^ and Cl^−^ on plant tissue are highly variable [3, 4]. In saline environments, the buildup of Na^+^ is a key risk factor that can cause specific ion toxicity, adversely affecting numerous crop species [5–7]. The inability of transporters to distinguish between Na^+^ and K^+^ due to their similar hydrated ionic radii can lead to excessive Na^+^ accumulation in plant cells, disrupting vital cellular functions [3]. Moreover, high levels of Na^+^ in the soil can also adversely affect the absorption of key minerals such as calcium (Ca^2+^) and magnesium (Mg^2+^) [8]. However, in certain contexts, low concentrations of Na^+^ may be beneficial to some non-halophyte plants, serving as a useful trace element [9]. High levels of Cl^−^ in soil, on the other hand, can reach toxic levels within cells, hampering plant growth and development. Excessive Cl^−^ is also linked to the reduction of photosynthetic capacity through the degradation of chlorophyll, which can damage the photosystem II (PSII) reaction centers [4]. Despite its potential downsides, Cl^−^ is also recognized as a micronutrient that is essential in small amounts for optimum plant yield and quality [10].

In salinity stress research, the concentration of Na^+^ in plant tissues is often used as a measure of the plant's tolerance or sensitivity. While Na^+^ is a common focus, Cl^−^ levels within plant tissues are less frequently examined [4, 11, 12]. However, high Cl^−^ concentrations are commonly found in plants subjected to saline conditions. The potential toxicity of high Cl^−^ levels and their impact on salt stress response merits further investigation, as it adds significance in understanding plant salt tolerance. Different plant species exhibit varying sensitivities to Na^+^ and Cl^−^, with some such as soybean and rice being particularly sensitive to Na^+^ [13, 14], while others like *Vicia faba* L. are more affected by Cl^−^ toxicity [4, 15]. In the case of onions, it has been observed that plants subjected to NaCl stress accumulate significant amounts of both Na^+^ and Cl^−^ [16]. Although onions are known to be sensitive to salinity [17], the distinct impact of Na^+^ versus Cl^−^ ions in their response to salt stress is not fully understood. A study by Romo-Pérez et al. [18] noted that moderate Na^+^ levels, resulting from Na_2_SO_4_ treatment, exerted a negligible effect on onion metabolism. This raises the possibility that Cl^−^ plays a more prominent role in onion sensitivity, particularly in scenarios where NaCl is the source of salinity stress.

Plant adaptation to salinity stress represents a complex interplay of metabolic and ionic responses, particularly involving Na^+^ and/or Cl^−^. Omics technologies, such as metabolomics, in conjunction with elemental analysis, offer an unprecedented opportunity to decode these intricate relationships. Despite the prevalence of such integrative approaches in model plants like *Arabidopsis* [19], the focus has been disproportionately on Na^+^, often at the expense of a thorough investigation into Cl^−^'s role. Our investigation seeks to harness the combined strengths of metabolomics and elemental analysis to elucidate the complex response mechanisms of plants subjected to saline environments. Shifting away from the one-sided sodium focus of prior studies, this research endeavors to explore the dual impact of Na^+^ and Cl^−^ ions, presenting an integrated view of the physiological adaptations in onions following salt exposure. To our knowledge, this represents the first comprehensive effort to apply this dual-methodological approach to analyze the initial response patterns of onion plants to Na^+^ and Cl^−^ accumulation post-salt treatment.

This investigation entailed a detailed examination of onion metabolism, focusing on amino acids, organic acids, and sugars, following treatments with KCl, K_2_SO_4_, Na_2_SO_4_, and NaCl. The treatments were administered to two distinct onion varieties with the intention of keeping ionic concentrations at equimolar levels. This stepwise application was designed to elicit early physiological and metabolic responses without causing sodium or chloride toxicity and plant death. This study introduces two novel focal points previously underexplored: the unique responses of onion leaves and bulbs to salinity and their specific reactions to Na^+^ and Cl^−^ ions. This organ-specific approach enhances our understanding of how different plant organs uniquely respond to elevated salinity. Additionally, the study examines both the individual and combined effects of Na^+^ and Cl^−^ within the metabolic and physiological contexts of onions. This dual-ion perspective is crucial for a more comprehensive understanding of the ionic impacts on the onion metabolome, particularly as onions initially experience saline conditions. The findings from this research aim to answer pivotal questions: Firstly, what are the initial metabolic indicators that manifest in onion plants under salinity conditions? Secondly, is there a greater sensitivity to Cl^−^ than to Na^+^ in onion plants? The findings shed light on these critical questions, thereby enhancing our knowledge of how plants regulate ions and adapt to stress induced by salinity.

## Materials and methods

### Plant cultivation, fertilization, salt application, and sample preparation

**Fig. 1:**
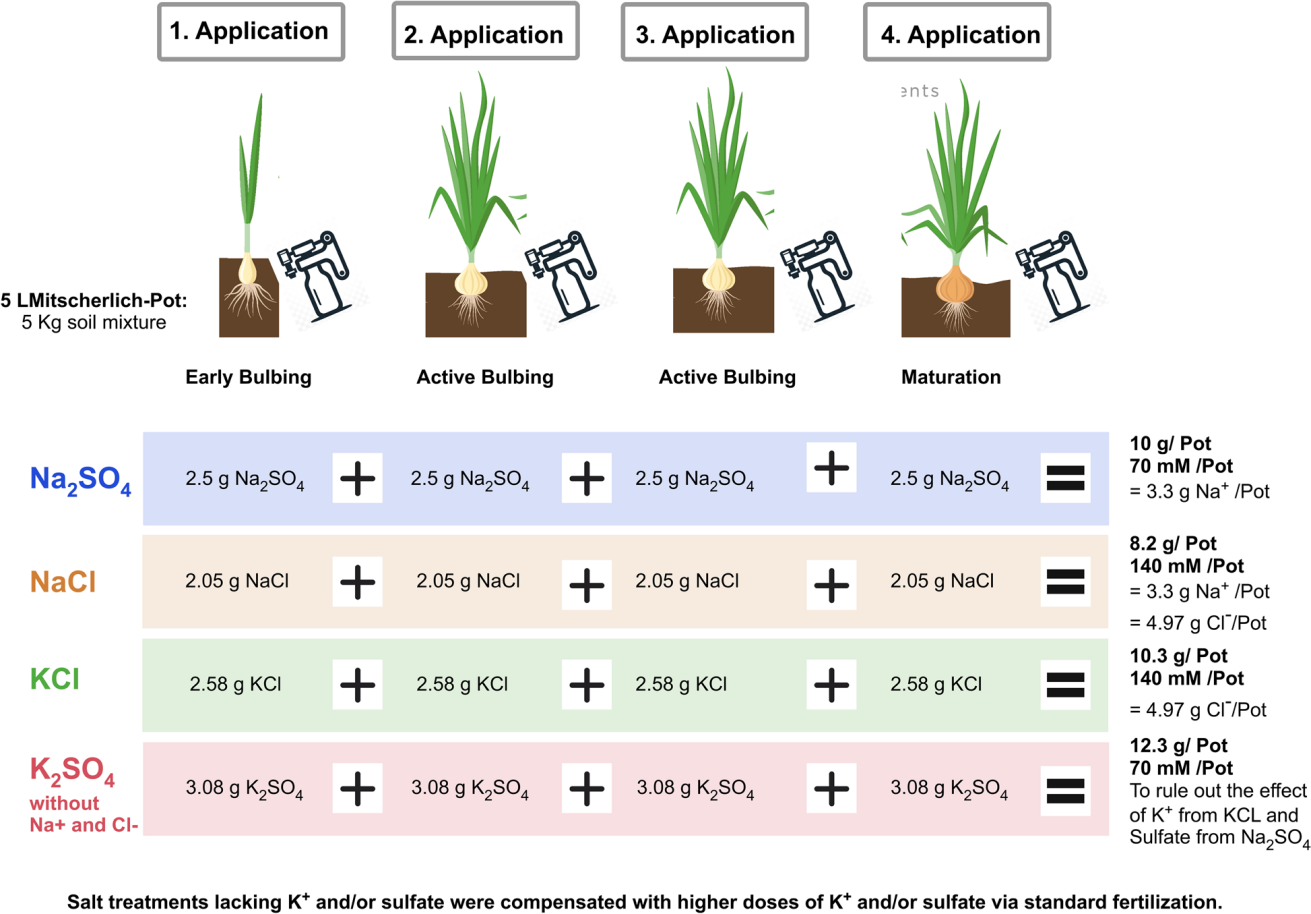
Salt treatment applications during the onion growing season. The data shown in the figure represent the amounts applied per Mitscherlich pot (5 kg soil mixture), with salts pre-dissolved in water. Each treatment was applied in four stages: early bulbing, active bulbing (twice), and maturation. Salt treatments lacking K^+^ and/or sulfate were compensated with higher doses of K^+^ and/or sulfate via standard fertilization. Sample size: *n* = 5

In the spring of 2020, the landrace 'Birnförmige' (or Birnenförmige) and the hybrid 'Hytech F1' onion seeds, obtained from Sativa e.V. and Bejo Seeds respectively, were cultivated in a greenhouse set to day/night temperatures of 18 °C/25 °C, with exposure to natural light cycles. After six weeks, the seedlings were transplanted into Mitscherlich pots with a 1:1 mixture of loam and sand, with four seedlings per pot, and later exposed to the outdoor environment, at coordinates 48°42′29.149" N, 9°12′42.25" E. The soil's pH was adjusted to 7.0 with the addition of 5% (w/w) sour turf. Base fertilization per pot was provided with 1 g of Mg(NO_3_)^2^, 1 g of Ca(NO_3_)^2^, 2 g of NH_4_H_2_PO_4_, and 0.3 g of Fetrilon combi micronutrient solution (AgNova Technologies). For pots not receiving potassium and sulfate from salt treatments, 2 g of K_2_SO_4_ was added to maintain the necessary levels, emphasizing the study's focus on the effects of Na^+^ and Cl^-^ as opposed to K^+^ and SO_4_^2-^. The pre-dissolved in water base fertilization was applied in two stages: 50% at the time of transplantation and the remaining 50% one month later. 

The study employed a completely randomized design with five replicates (*n*=5) for each treatment and variety. Salt treatments were administered using equimolar concentrations of Na^+^, K^+^, and Cl^-^ derived from four salts: Na_2_SO_4_ at 70 mM per pot, KCl at 140 mM per pot, NaCl at 140 mM per pot, and K_2_SO_4_ at 70 mM per pot. The salts, pre-dissolved in water, were applied in a staged manner at four critical growth phases of onion bulbing: early bulbing, twice during active bulbing, and once at maturation (Figure 1). Each application involved dispensing the solutions using a 100 mL tilt measure to ensure precise and uniform distribution. Irrigation was applied based on plant needs. Initially, the irrigation intervals were set to once a day before and during the early bulbing and maturation stages. During the active bulbing stage, when water demand and summer temperatures were higher, irrigation was increased to twice per day. The run-off was collected and channeled back to the reservoir of the Mitscherlich pots to be reused for further irrigation, in order to minimize water, salt, and mineral losses applied through fertilization and treatments.

At harvest, plant foliage was separated, shock-frozen in nitrogen, and prepared for metabolic and mineral analysis. Soil samples were oven-dried at 100 °C for 48 hours for mineral and conductivity assessments. Bulbs were cured, weighed, and sectioned into wedges, which were pooled, shock frozen, freeze-dried, and then powdered for metabolic, mineral, ion, and antioxidant analysis. The remaining bulb halves were blended for analyses of pyruvic acid, non-structural carbohydrates, and dry matter content.

### Potassium, magnesium, calcium, sodium, chloride and sulfate analysis

Cation concentrations (Na^+^, K^+^, Mg^2+^, Ca^2+^) were assessed using approximately 50 mg of freeze-dried leaf material and 100 mg of bulb and soil samples. These samples were digested in a solution of 8 ml 69% nitric acid and 4 ml hydrogen peroxide, using a microwave digestion system at 190 °C for 25 min (CEM, Mars 5, Matthews, USA). The resulting cation concentrations were then quantified through atomic absorption spectrometry using a 3300 series instrument (Thermo Fisher Scientific, Dreieich, Germany). Cl^−^ content was determined in 200 mg of freeze-dried leaf and bulb samples using a chloride meter (Model 6610, Eppendorf AG, Hamburg, Germany) following the methodology of Zhang et al. [20].

For analysis of total sulfur, 30 mg freeze-dried leaf and bulb and soil material were examined in a CNS elemental analyzer (Vario max CNS, Elementar Analysensysteme GmbH, Hanau, Germany). The values presented refer to dry weight (DW). Sulfate (SO_4_^2+^) levels in onion leaves and bulbs were quantified by processing 100 mg of freeze-dried samples. Each sample was heated in 700 µl of deionized water at 95 °C for 90 min at a stirring speed of 900 rpm. Post-heating, the samples were filtered, and sulfate content was measured using High-Performance Liquid Chromatography (HPLC) using an VWR/HITACHI Chromaster 5000 chromatograph (VWR International GmbH, Bruchsal, Germany). However, due to HPLC's sensitivity range, SO_4_^2+^ levels in bulbs weren't measurable. The sulfate concentrations in the leaves varied widely, with some near the detection threshold, and for this reason, these values are omitted from the paper.

### Quality parameters, targeted and untargeted metabolite analysis

The quantification of dry matter, total soluble solids, non-structural carbohydrates, pungency (pyruvic acid), and antioxidant activity in onion bulbs followed the protocols conducted by Romo-Pérez et al. [21]. Dry matter content was assessed by oven-drying 20 g of homogenized samples at 65 °C for 48 h and then at 105 °C for an additional 3 h. Non-structural carbohydrate analysis employed the Official Analytical Chemist (AOAC) methodology alongside the Megazyme fructan assay kit (K-FRUC, Megazyme, Ireland) with p-hydroxybenzoic acid hydrazide (PAHBAH), as per McCleary et al. [22]. Onion pungency measurement was performed using Anthon and Barret [23] improved technique and the background pyruvic acid method by Yoo and Pike [24]. Antioxidant capacity in the onion bulbs was determined via the 2,2-diphenyl-1-picrylhydrazyl (DPPH) spectrophotometric method, as described by Brand-Williams et al. [25]. Firmness was measured using a digital penetrometer (PCE-PTR 200, Meschede, Germany), and total soluble solids were gauged with a handheld refractometer (Schneider 161,030, Albertshausen, Germany). For quercetin concentration analysis, we used 100 mg of freeze-dried onion bulb samples, which were suspended in 80% ethanol and shaken for three hours at room temperature using a vertical rotator (Grant instruments, PTR-25 360°). The mixture was then centrifuged at 14000 rpm for 10 min and the supernatants were filtered through a PTFE filter, with the filtrate stored at -80 °C for subsequent measurements. Analysis required standards of quercetin-4’-O-glucoside, quercetin 3–4’O-diglucoside, and quercetin. HPLC analysis of all extracts was carried out using an VWR/HITACHI Chromaster 5000 chromatograph (VWR International GmbH, Bruchsal, Germany) equipped with a solvent delivery system, an auto-sampler, a diode array detector set at 360 nm, and a Chrommaster system manager data acquisition system (Hitachi High-Technologies Corporation, Tokyo, Japan). Flavonoids were separated on a Zorbax Eclipse Plus C-18 column (250 mm × 4.6 mm, part number 959990–902) with a particle size of 5 μm (Agilent, Waldbronn, Germany) protected with an Agilent Zorbax Eclipse Plus C-18 narrow bore guard column (2.1 × 12.5 mm, part number 821125–836). The column was maintained at 25 °C. The mobile phase consisted of 0.1% trifluoroacetic acid (TFA) in water (solvent A) and methanol (solvent B). The gradient elution program was set as follows: 0–10 min, 20% B; 10–15 min, 20–80% B; 15–22 min, 80–20% B. The flow rate was 0.8 mL min − 1, and the injected volume was 10 μL. Quercetin flavonols were quantified through comparison with the respective calibration curves. Chromatographic analysis of each replicate sample was repeated twice, and the average peak height was used in calculations.

Untargeted metabolite analysis including data processing was conducted as described by Romo-Pérez et al. [18], with some modifications: In case of both leaf and bulb samples, 20 mg of freeze-dried material were extracted twice with 750 µL of methanol. The volumes of the derivatization reagents were 20 µL of methoxylamine-hydrochloride in pyridine (20 mg/mL) and 50 µL of MSTFA (without TMCS). The ^1^D column was a Rxi-5SilMS (^1^L = 20 m plus 5 m of an integrated pre-column, ^1^d_c_ = 0.18 mm, ^1^d_f_ = 0.18 µm; Restek, Bellefont, USA). The GC temperature ramp was 90 °C → 3,5 °C/min → 200 °C → 6.0 °C/min → 345 °C (hold 1.40 min), leading to a run time of 57 min. The initial column head pressure was 160 kPa. The split ratio was 1:3 (hold 0.5 min) → 1:30 (hold until 4 min) → 1:10 (hold until 15 min) → 1:3 (hold until end of run), the injection volume 1 µL. The ion source was operated at a temperature of 250 °C and the MS interface at 290 °C. The modulation period was 2.2 s.

### Statistical analysis

The untargeted GC × GC–MS metabolomics dataset was analyzed in two stages using JMP 15.1.0 (SAS Institute Inc., Cary, NC, 1989–2019). Data matrices for all varieties collectively and for each variety individually were initially prepared. Variables with more than or equal to 25% non-detects were excluded and replaced with random numbers ranging from 5,000 to 10,000. These processed data matrices included leaf and bulb samples. Statistical analyses were performed with R version 4.2.0 (https://www.r-project.org) and MetaboAnalyst 6.0 (https://www.metaboanalyst.ca). Two-way ANOVA, followed by Tukey HSD post-hoc tests, was conducted to assess the effects of variety and treatment. Linear models were utilized for data analysis, with *p*-values < 0.05 denoting statistical significance. Principal Component Analysis (PCA) was employed to visually represent the characteristics of the varieties and the impact of treatments, standardizing and centering data before analysis with the R package "factoextra" [26]. Only the values of identified metabolites from the untargeted analysis were included in the matrix for PCA. Additional figures were generated using bar plots, heatmaps, and correlation figures with the "ggplot2" package [27], heatmaps with "pheatmap" [28], and Euler diagrams with "eulerr" [29]. For univariate analysis of treatment effects, ANOVA with False Discovery Rate (FDR) adjustment was applied to each variety and sample matrix separately using JMP's Response Screening platform. Compounds of potential importance were visually selected from "FDR LogWorth vs. Effect Size" plots, with significance confirmed by Tukey-HSD post-hoc testing. The correlation diagram included metabolites with a *p*-value < 0.01. Additionally, a threshold line at 0.7 was marked to emphasize the most prominent components that correlated more than 70% positively or negatively with Na^+^ or Cl^−^, respectively.

## Results

### Ion concentration in onion plants

As Romo-Pérez et al. [18] previously described, moderate sodium levels provided by sodium sulfate (1.3 g of sodium per 5 L pot) had only minimal effects on onion plant physiology and metabolomic profiles. Building on these findings, our research investigated higher sodium levels to determine the concentration at which Na^+^, Cl^−^, or their combination starts to produce initial effects on the metabolomic profile without adversely affecting onion bulb development. To prevent over toxicity or signs of senescence such as necrosis or chlorosis, treatments were systematically applied in four increments during the onion's bulbing stages, maintaining the equimolarity of the salt ions with each addition (Fig. 1). The ultimate concentrations administered were 3.3 g of Na^+^ for both NaCl and Na_2_SO_4_ treatments, and 5 g of Cl^−^ for treatments involving NaCl and KCl per pot (5 kg soil mixture). The salt K_2_SO_4_ served as a comparative salt to rule out the distinct impacts of potassium from KCl and sulfate from Na_2_SO_4_, thereby emphasizing the specific effects of Na^+^ and Cl^−^.

**Fig. 2:**
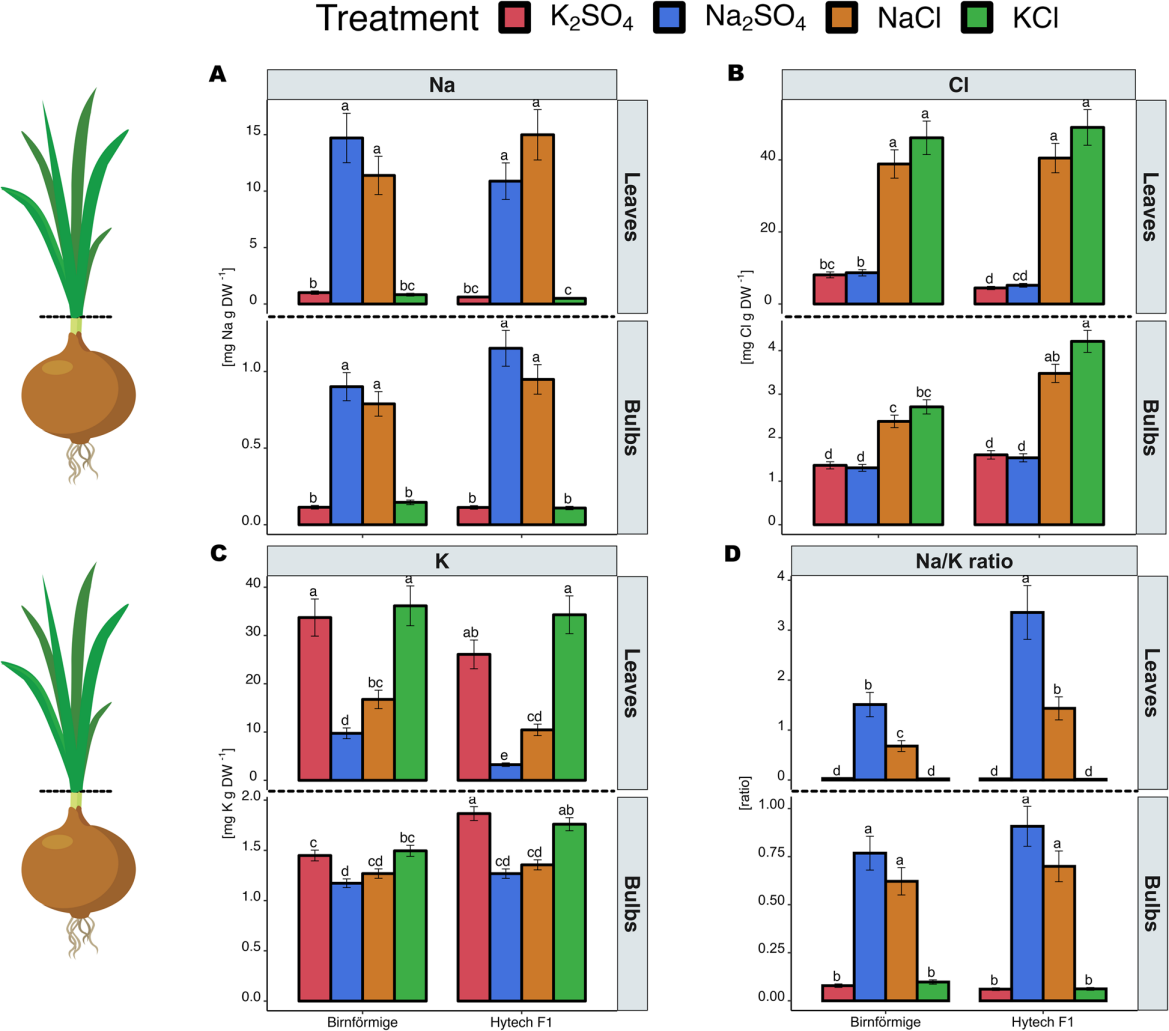
Ion concentrations in leaf and bulb, of two varieties, landrace Birnförmige and hybrid Hytech F1 of *Allium cepa* L. **A** Sodium concentration in leaf and bulb of onions. **B** Chloride concentration in leaves and bulbs of onions. **C** Potassium concentration in leaves and bulbs of onions and **D** Na^+^/K^+^ ratio in leaves and bulbs of onion plants. Data are mean ± SE. Significant test by Tukey’s HSD (*p* < 0.05), after two-way ANOVA, indicated by different letters. (*n* = 5)

Figure 2A shows the effect of NaCl and Na_2_SO_4_ treatments on Na^+^ concentration in leaf and bulb tissues of the two onion varieties, Hytech and Birnförmige. The results indicate a substantial increase in Na^+^ concentration in both varieties. In particular, Hytech exhibited in leaf tissue an increase from 0.5 mg Na g DW^−1^ in treatments without Na^+^ to 15.3 mg Na g DW^−1^ in treatments with Na^+^, and from 0.11 mg Na g DW^−1^ to 1.2 mg Na g DW^−1^ in bulb tissue. For Cl^−^ (Fig. 2B), both varieties showed a significant increase in Cl^−^ accumulation under KCl and NaCl treatments, with Cl^−^ levels rising from 4.5 mg Cl g DW^−1^ to 50 mg Cl g DW^−1^ in leaves, and from 1.5 mg Cl g DW^−1^ to 4.2 mg Cl g DW^−1^ in bulbs. Notably, there was a considerable disparity between the two onion varieties regarding Cl^−^ accumulation, with Hytech accumulating significantly more Cl^−^ than Birnförmige in the bulbs. In Fig. 2C it is evident that NaCl and Na_2_SO_4_ treatments resulted in a noteworthy decrease in K^+^ concentrations in both leaves and bulbs for both varieties. Notably, Na_2_SO_4_ had a greater impact on reducing K^+^ levels compared to NaCl, particularly observed in variety Hytech. The Na^+^/K^+^ ratio results show clear differentiation between the two onion varieties, Hytech and Birnförmige. In Hytech, the Na^+^/K^+^ ratio in leaf tissue ranged from 0.03 in the K_2_SO_4_ treatment to 1.48 in the NaCl treatment and 3.43 in the Na_2_SO_4_ treatment. In contrast, Birnförmige exhibited lower Na^+^/K^+^ ratios, ranging from 0.03 in the K_2_SO_4_ treatment to 0.73 in the NaCl treatment and 1.53 in the Na_2_SO_4_ treatment. These results demonstrate a clear differentiation in Na^+^/K^+^ balance regulation between the two varieties, with Birnförmige consistently showing a more effective regulation compared to Hytech, particularly under saline conditions (Fig. 2D). Subsequent analyses were performed to evaluate the concentrations of magnesium (Mg^2+^) and calcium (Ca^2+^) in the plant tissues, as detailed in Supplemental material, figure S1. These assays revealed that Cl^−^ and Na^+^ administrations were not detrimental to the concentrations of Ca^2+^ and Mg^2+^; in fact, a positive association between the levels of these ions and the concentrations of Ca^2+^ and Mg^2+^ was observed.

As detailed in supplementary material, figure S1, our data revealed a marked increase in total sulfur (S) content in the leaves of onion plants, with the Birnförmige variety showing a pronounced increase following treatments with Na_2_SO_4_ and K_2_SO_4_. Conversely, no significant increase in sulfur was detected in the bulbs of either the Hytech or Birnförmige varieties. It is noteworthy that standard fertilization procedures were followed, ensuring an adequate supply of essential nutrients, including K^+^, Ca^2+^, Mg^2+^, NO_3_^−^, PO_4_^3−^, and SO_4_^2−^, to avoid deficiencies in key minerals and companion ions.

### Effects of different salt treatments on physiological and quality parameters in onion plants

**Fig. 3:**
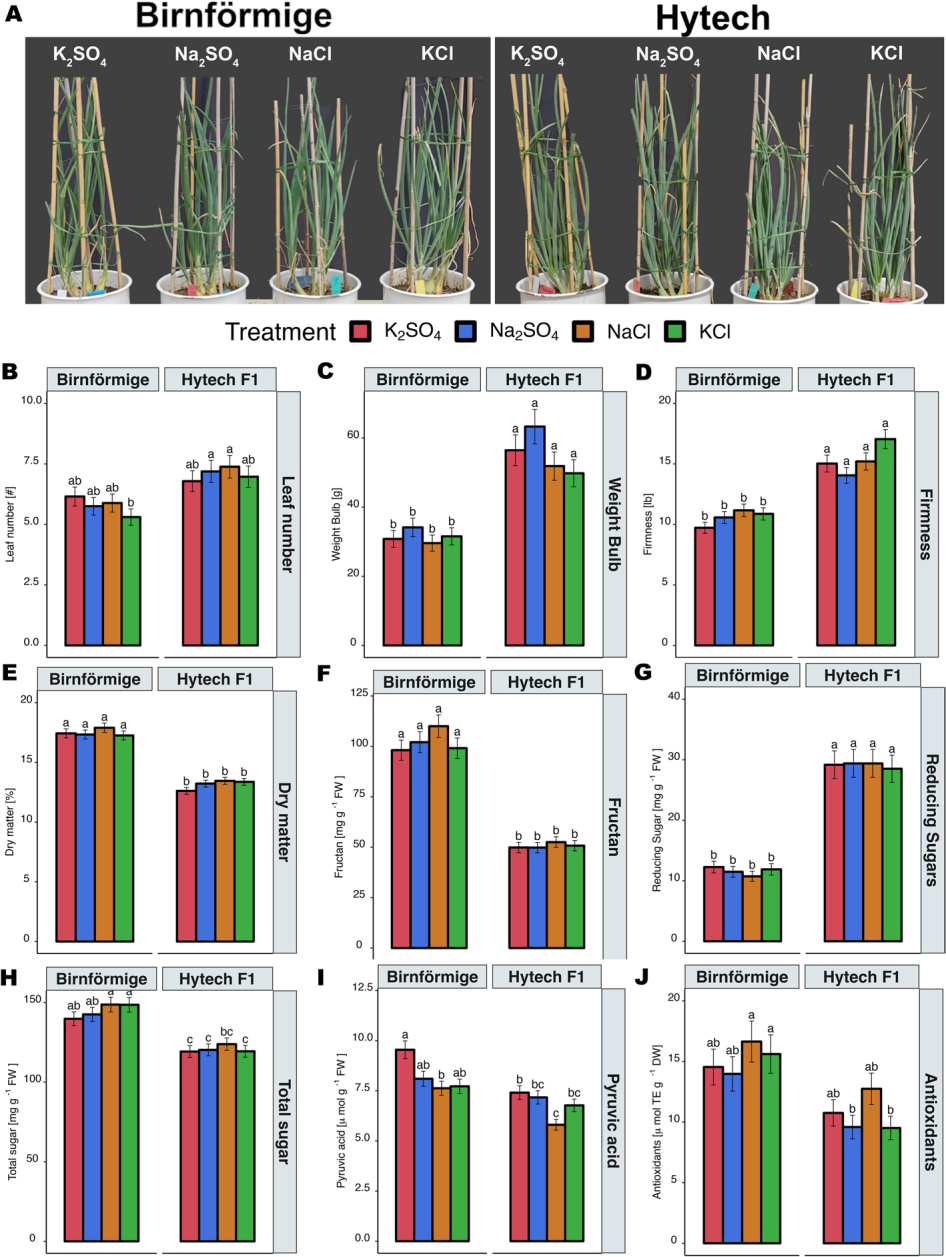
Physiological parameters of onion plants, and absolute concentrations of relevant compounds in the bulbs of the two varieties. **A** Onion plants (4 plants/pot) two weeks after the final application of Na_2_SO_4_, NaCl, KCl, and the variant without Na^+^ and Cl^−^ (K_2_SO_4_). **B** Number of leaves per plant, **C** Average weight of onion bulbs from 4 plants per plot, **D** Firmness of onion bulbs, **E** Dry matter of onion bulbs, **F** Fructan concentration in onion bulbs, **G** Concentration of reducing sugar in onion bulbs, **H** Total sugar in onion bulbs, **I** Pyruvic acid concentration (onion pungency) in onion bulbs, **J** Antioxidants (antioxidant activity). Data are presented as mean ± standard error (SE). Statistical significance determined by Tukey's Honest Significant Difference (HSD) test (*p* < 0.05) after a two-way ANOVA, is indicated by differing letters above the bars. Sample size *n* = 5

The current study aimed to investigate the early metabolic responses of onions to elevated salinity conditions by applying non-toxic salt concentrations. This approach enabled us to capture the initial metabolic, ionic, and osmotic changes induced directly by Na^+^ and Cl^−^ ions without triggering secondary physiological effects or salt toxicity symptoms, which could obscure the direct impacts of these ions on the onion metabolome and physiological traits. Given that the transition from early bulbing to active bulbing in onions is highly sensitive to stress, exposure to elevated levels of Na^+^ and/or Cl^−^ during these stages often halts bulb development, leading to stunted growth. To prevent disruptions in bulb formation, toxicity treatments were deliberately excluded. Instead, the study focused on monitoring the early physiological and metabolic responses of onions to increasing salinity levels while maintaining Na^+^ and Cl^−^ concentrations below toxic thresholds. As a result, all onion plants, irrespective of variety and treatment, displayed no visible signs of stress and stunted growth (Fig. 3A). Furthermore, there were no significant differences in leaf number between the treatments (Fig. 3B), with Birnförmige variety averaging 5 -7 leaves and Hytech variety averaging 6—8 leaves during active bulbing after complete salt application.

Subsequent analyses of plant fresh weight, firmness, dry matter content, and non-structural carbohydrates (Fig. 3C-H) indicated similar values across the treatments, supporting the successful avoidance of toxic stress in the plants. Among the parameters measured, pyruvic acid, a marker for onion pungency, was the only one that displayed a significant reaction to the treatments Fig. 3I. Both hybrid Hytech and landrace Birnförmige exhibited similar responses to the NaCl treatments, resulting in a slight, but significant decrease in pyruvic acid levels. For the hybrid Hytech, values ranged from 5.8 μmol g^−1^ FW (fresh weight) for NaCl to 7.4 μmol g^−1^ FW for K_2_SO_4_, while for landrace Birnförmige, values ranged from 7.6 μmol g^−1^ FW (NaCl) to 9.6 μmol g^−1^ FW (K_2_SO_4_). Interestingly, there was a slight increase in antioxidants following the NaCl treatments shown in Fig. 3J, indicating an opposite behavior compared to pyruvic acid. For the Hytech variety, values ranged from 10.8 μmol g^−1^ FW (K_2_SO_4_) to 12.8 μmol g^−1^ FW (NaCl), while for Birnförmige variety, values ranged from 15.0 μmol g^−1^ FW (K_2_SO_4_) to 16.7 μmol g^−1^ FW (NaCl). Quercetin concentrations did not exhibit significant increases or decreases due to the treatments according to supplementary data, figure S2. However, there was a discernible difference between varieties, with hybrid Hytech containing higher quercetin levels than the landrace Birnförmige.

Despite the treatments having little to no effect on the measured quality parameters, there were significant differences observed between the two varieties. On average, the hybrid Hytech variety produced more leaves and had larger and firmer onion bulbs compared to Birnförmige. However, the landrace Birnförmige exhibited higher values for dry matter content, fructan, total sugar, pyruvate, and antioxidant concentration.

### General metabolic response to salt treatments in onion plants

**Fig. 4:**
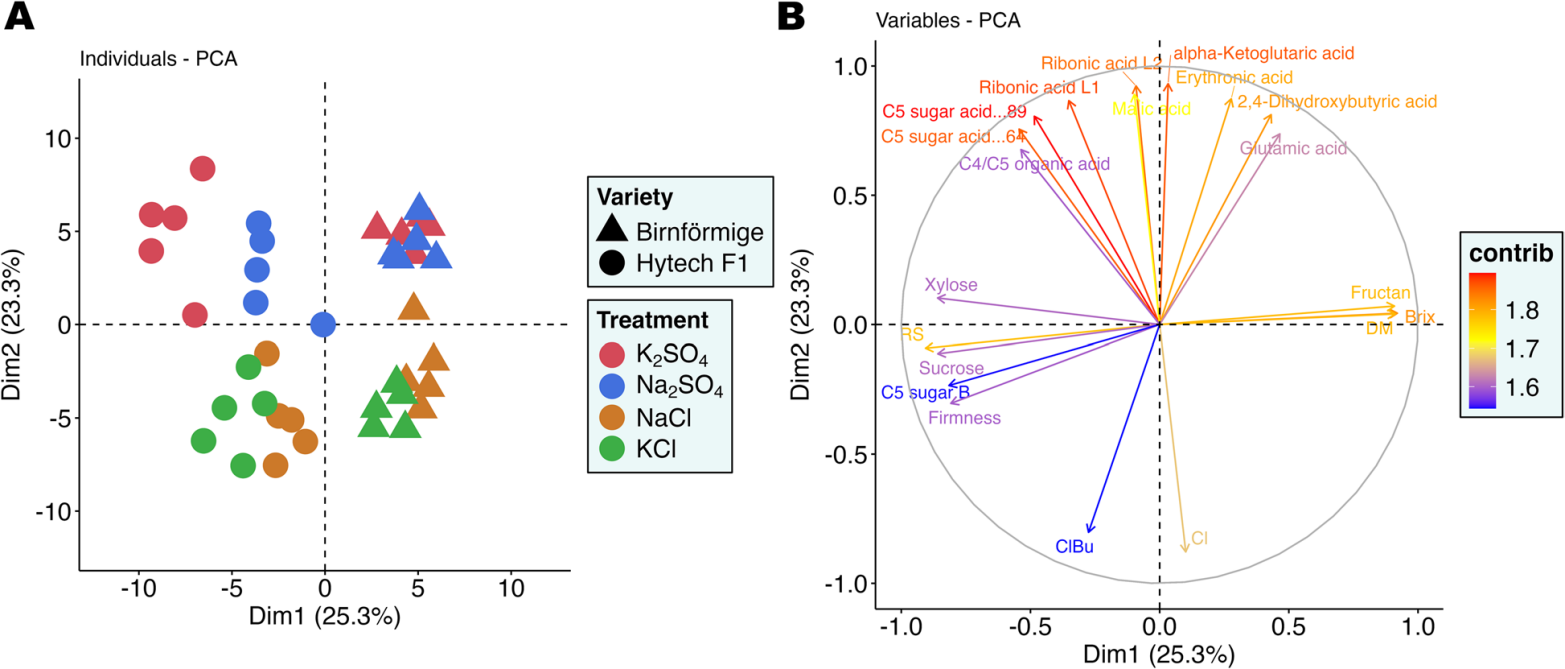
Response of whole onion plant (including data of bulb and leaf) to salt treatments. Data were visualized by principal component analysis (PCA) of the first two components. This PCA captures 48.61% of the dataset's total variance, indicating a significant proportion of the data's variability. **A** PCA scores plot. Hytech F1 (●), Birnförmige (▲). Treatments are represented in different colors: K_2_SO_4_, Na_2_SO_4_, NaCl and KCl. **B** PCA loadings plot displaying the overall contribution of measured variables to the first two principal components. Variables are marked in red- blue and connected by vectors to their relative importance on the Dim1 and Dim2 axes, demonstrating their impact on the variance explained by each component. The length and direction of the vectors suggest how each variable correlates with principal components and with one another. Variables are colored based on their weight of the contribution to the two axes shown. (*n* = 5). The plot encompasses all data from both targeted and untargeted metabolomics analyses; however, only identified and affected metabolites from untargeted metabolomics are considered in the PCA. The top 20 most influential variables are emphasized in the loadings plot

In the context of plant stress, the regulation of metabolites is crucial for maintaining osmotic balance. Principal Component Analysis (PCA) visualized the effects of different ions and salts on the metabolomic profiles of two onion varieties. According to the PCA plot (Fig. 4A), which includes data of both varieties independent of the plant organ, the majority of the metabolic variation, accounting for 48.6% of the total, was associated with the type of onion variety. The X-axis, which represented 25.3% of the variance, clearly distinguished between the landrace Birnförmige and hybrid Hytech. In terms of the Y-axis, the levels of Cl^−^ (Cl, ClBu) played a pivotal role in segregating the data into two distinct clusters: one representing treatments without chloride (K_2_SO_4_ and Na_2_SO_4_) positioned on the graph's higher segment, and another representing treatments with increased chloride content (NaCl and KCl) appearing on the lower segment as illustrated in Fig. 4B. In contrast to the prominent role of Cl^−^ as indicated in the loading plot (Cl), the influence of sodium on the metabolic profile was relatively subdued, not ranking within the top 20 influential compounds in the loading plots.

In addition to NaCl and KCl treatments, potential impact of Na_2_SO_4_ and K_2_SO_4_ on sulfur metabolism in onion plants was considered. To clarify these effects, we also analyzed the response of metabolites involved in sulfur metabolism. Our observations indicated that treatments containing sulfate (Na_2_SO_4_ and K_2_SO_4_) did not significantly alter the concentrations of metabolites like methionine, cysteine, O-acetylserine, S-Methylcysteine, neither the glycine and serine pools, thus these factors did not appear among the top 20 influential variables (Fig. 4B). Furthermore, sulfur’s involvement was not evident in the loading plots of the dataset.

### Specific metabolic response of the salt treatments on two different onion varieties

**Fig. 5:**
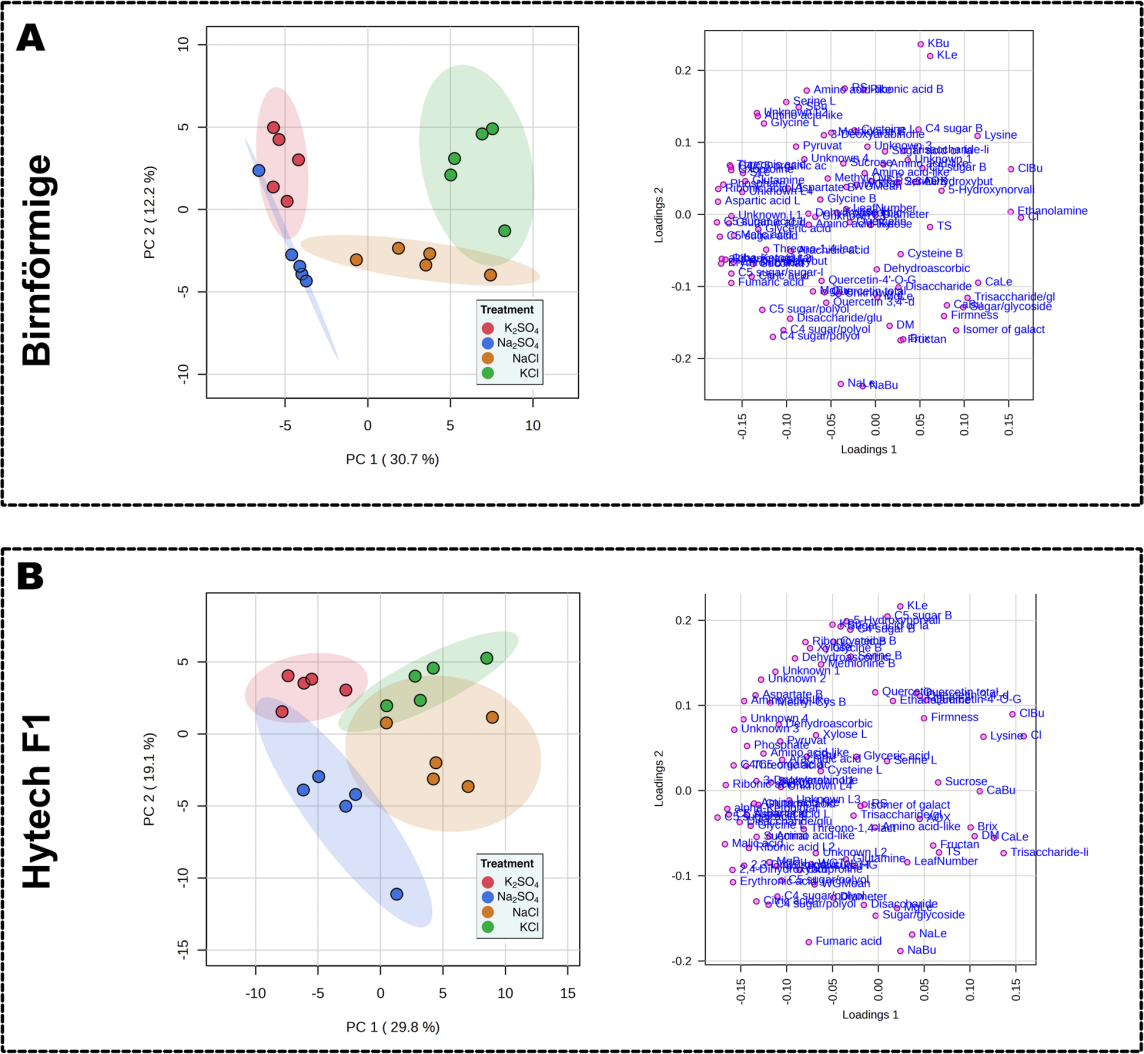
Variety specific differences explained with PCAs and loading plots of the both varieties separately. **A** PCA (Principal Component Analysis) scores, and loading plots for the onion variety Birnförmige. The PCA and loading plots show that 42.9% of the dataset's variance is captured, reflecting a significant amount of the data's variability. **B** Similar PCA scores, and loading plots for Hytech. Here, the PCA captures 48.93% of the dataset's total variance, also indicating substantial data variability. This variety's data grouping patterns can be observed in the dendrogram. Treatments are represented in different colors: K_2_SO_4_, Na_2_SO_4_, NaCl and KCl; (*n *= 5)

Further investigation into the metabolic alterations due to specific ion/salt concentrations in each onion variety was achieved by performing separate one-way ANOVA tests, followed by PCA analysis. The PCA outcomes (Fig. 5A-B) revealed two principal components that explained 48.9% of the variance for Hytech and 42.9% for Birnförmige. Both varieties had a robust response to increased levels of Cl^−^ (Cl, ClBu). For Birnförmige, as shown in Fig. 5A, elevated Cl^−^ levels significantly affected the X-axis distribution, with a positive correlation with ethanolamine and a negative correlation with many metabolites, especially organic acids. Na^+^ accumulation had a less pronounced effect (NaLe, NaBu), showing a negative correlation with K^+^ on the Y-axis. In the Hytech variety, as depicted in Fig. 5B, Cl^−^ negatively impacted as well a broad array of organic acids, and there was a higher sensitivity to increased Na^+^ levels, which affected not only K^+^ but also a range of other metabolites, including xylose and a specific C4 and C5 sugars.

### Specific metabolic response in leaves and bulbs of onion varieties

**Fig. 6:**
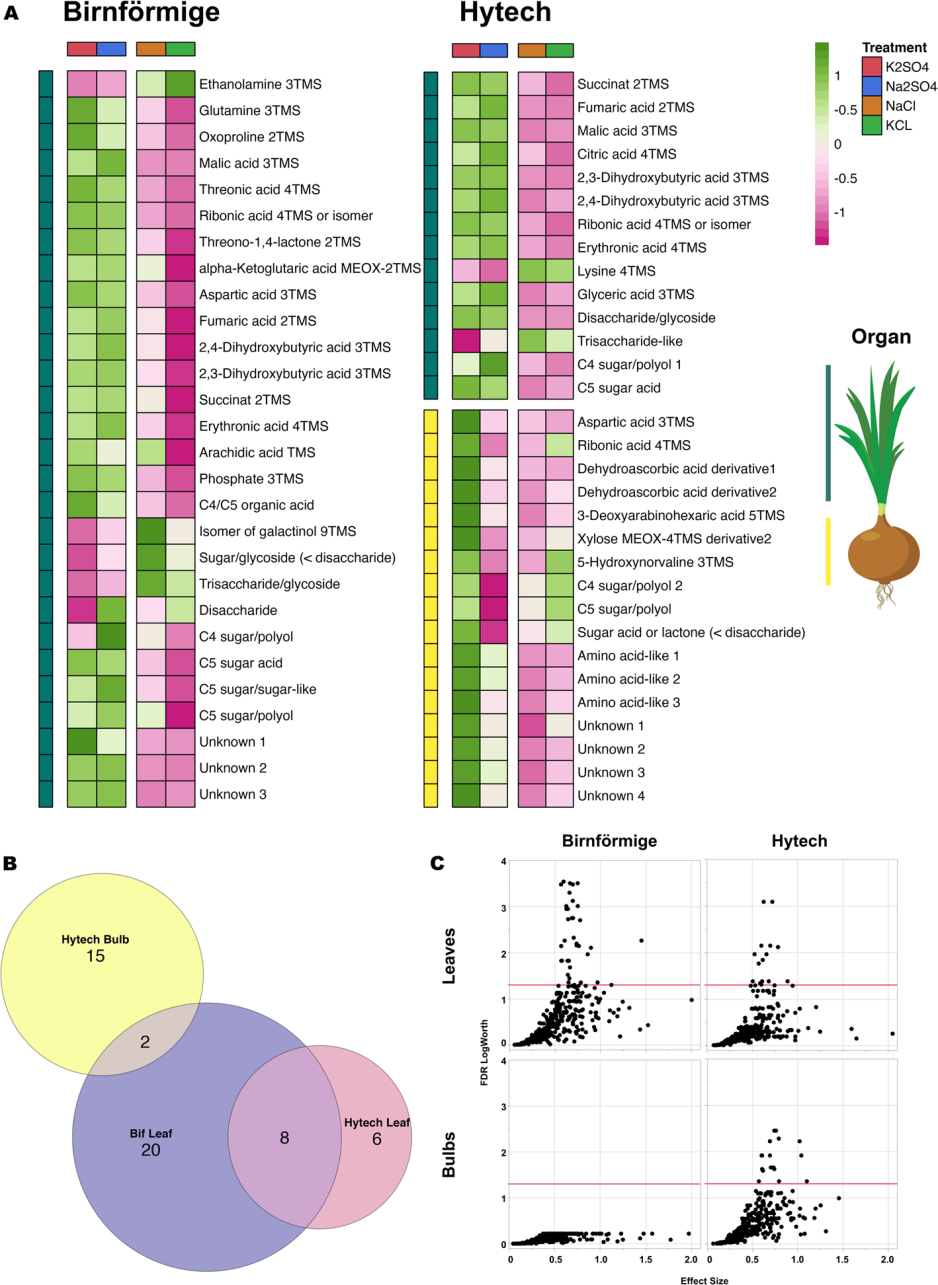
Metabolic profiling of Birnförmige and Hytech onion varieties under salt stress. **A** Metabolic Heatmap Analysis: Heatmap showcasing relative metabolite concentrations in Birnförmige (leaves) and Hytech (leaves and bulbs). Elevated metabolite concentrations are color-coded in green, whereas reductions are coded in pink. The intensity of color signifies the level of change, with statistical significance marked at *p* < 0.01. **B** Venn Diagram of Metabolite Changes: Diagram delineating the shared and exclusive metabolic alterations in the leaves and bulbs of Birnförmige and Hytech varieties post salt treatment, offering insight into unique and common stress responses. **C** ANOVA-Based Response Screening: Graphical representation of the Response Screening analysis from GC × GC–MS data. The plot displays "FDR LogWorth vs Effect Size" for individual metabolites, with each point depicting an analyte's variation. Significance is determined by the FDR LogWorth value (negative log-transformed *p*-value), with a threshold of p > 0.05 signifying substantial changes (above the red line at FDR Logworth = 1.3). Analysis incorporates relative untargeted metabolomics data

Both, the landrace Birnförmige and the hybrid Hytech exhibited some similar reactions to chloride salts. Treatments with Cl^−^ (NaCl and KCl) resulted in reduced levels of eight specific metabolites in the leaves: succinate, fumaric acid, 2,3-dihydroxybutyric acid, 2,4-dihydroxybutyric acid, malic acid, ribonic acid, a C5 sugar acid, and erythronic acid (Figs. 6A and 6B). However, notable differences between the two varieties emerged, particularly in the organ-specific responses to the treatments, as depicted in Fig. 6A. with increases and decreases in the levels of 28 metabolites in the leaves, without a corresponding change in the bulbs (Fig. 6C). Conversely, Hytech's leaves showed a considerable reduction in 14 metabolites in response to chloride salt treatments. In Hytech's bulbs, 17 metabolites, including xylose, ribonic acid, and a C4 sugar polyol, significantly varied not only with Cl^−^ but also with Na^+^-salt treatments.

**Fig. 7:**
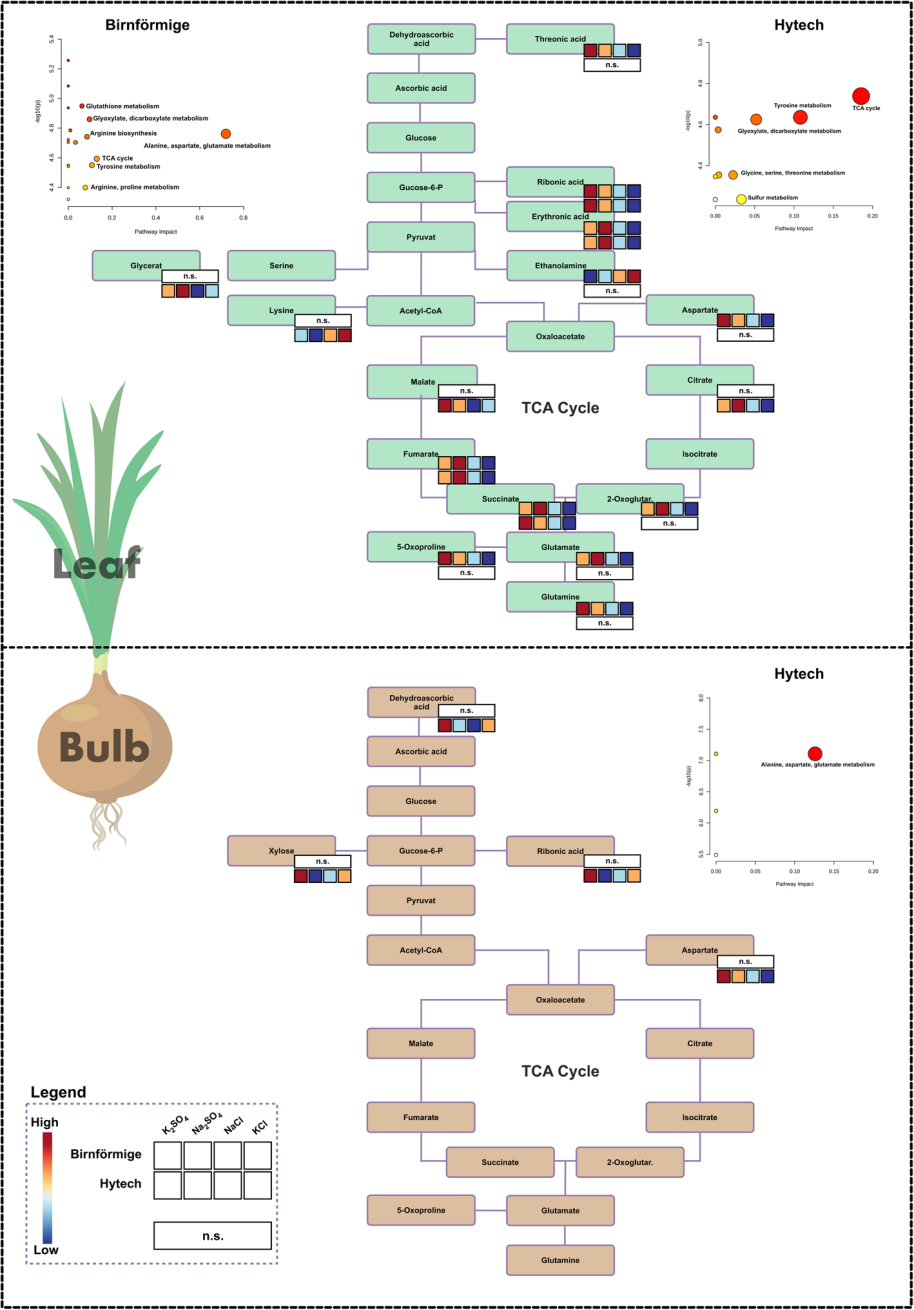
Illustrative overview of metabolites within the biosynthetic pathways of onions, comparing the Birnförmige and Hytech varieties. The top portion of the diagram represents leaf metabolites, and the bottom portion shows bulb metabolites impacted on the pathway. Metabolite fluctuations are color-coded: higher levels in red, lower levels in blue, and no significant change in white. Each rectangle in the diagram corresponds to a specific metabolite along the pathways, with a focus on the tricarboxylic acid (TCA) cycle. Accompanying the diagram, the smaller plots quantify the pathway's impact and the relative *p*-values, using circle colors and sizes to represent significance and impact: intense red and larger sizes denote high significance and pathway impact, indicating notable changes brought about by the treatments

Figure 7 highlights the effects of salt treatments on biosynthetic pathways in both varieties. In the leaves, there were pronounced alterations in organic acid levels, particularly those participating in the tricarboxylic acid (TCA) cycle. Chloride-rich treatments additionally lowered the levels of other metabolites such as ribonic acid and erythronic acid in both varieties. While the bulbs did not show a reduction in TCA cycle metabolites, the hybrid Hytech displayed decreased levels of aspartate under conditions high in Cl^−^. Conversely, Na^+^-rich treatments (Na_2_SO_4_, NaCl) notably reduced the levels of xylose, ribonic acid, and dehydroascorbic acid in the bulbs of the hybrid Hytech.

### Assessment of metabolic and ionic profiles in response to Na^+^ and Cl^−^ accumulation

To investigate the effects of Na^+^ and Cl^−^ accumulation on the metabolism under saline conditions and to pinpoint potential sites of metabolic regulation, we conducted a comparative analysis. This analysis involved correlating the concentrations of Na^+^ and Cl^−^ with the changes in metabolite levels and other ions in leaves and bulbs.

**Fig. 8:**
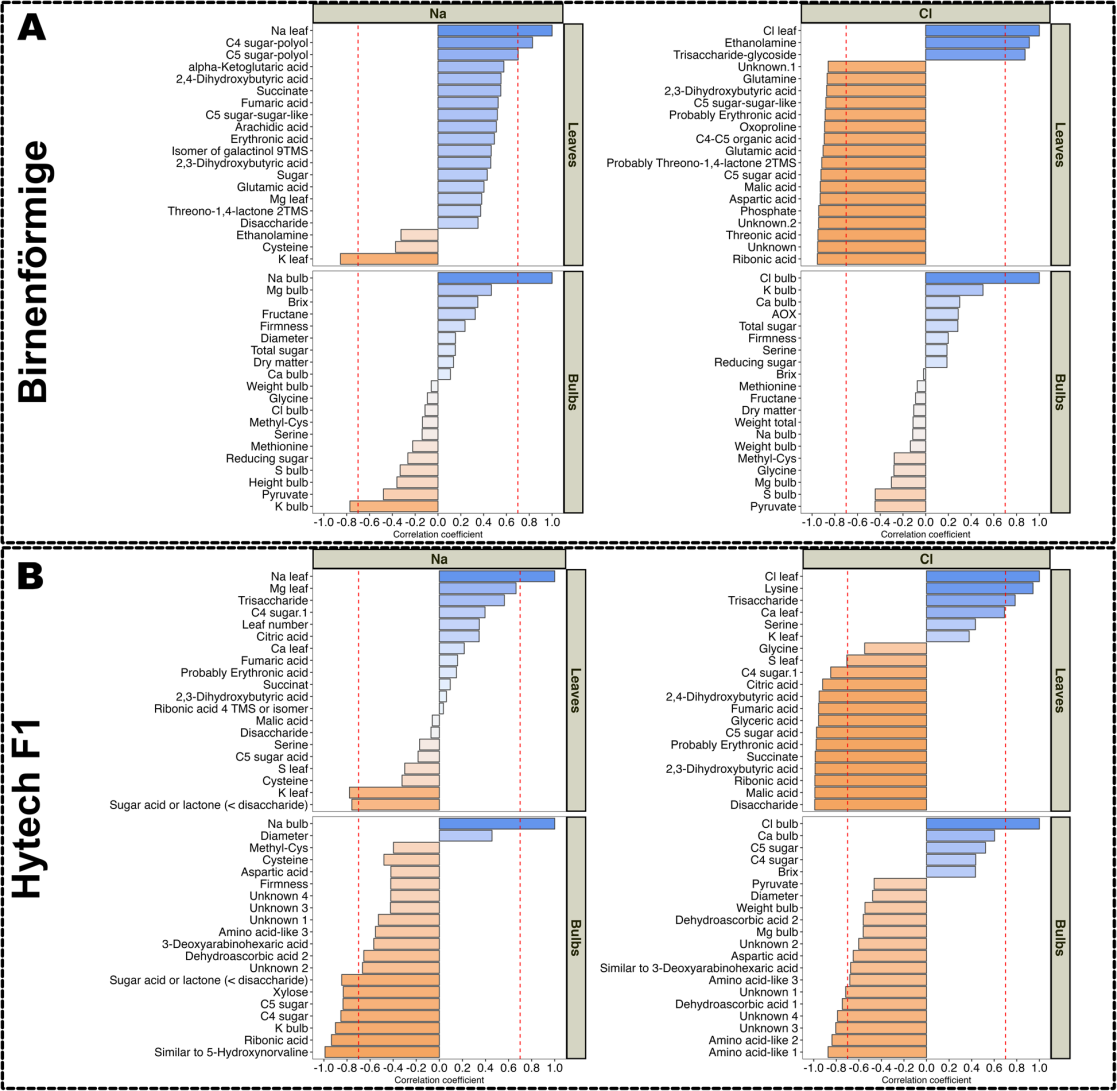
Relationship between sodium and chloride accumulation and the respective changes in metabolites and ions for two varieties of onions. **A** Birnförmige, leaves and bulbs. **B** Hytech, leaves and bulbs. Bar graphs representing the strength and direction of the correlation between the accumulated ions (Na^+^ on the left, Cl^−^ on the right) and various metabolites/ions. Blue bars indicate positive correlation, while orange bars indicate negative correlation with the respective ion. The color intensity indicates the level of statistical significance, with more vivid colors reflecting lower *p*-values. The figure also features a dashed red line indicating the threshold for a strong correlation r > .70

In the leaves of the landrace Birnförmige Na^+^ was positively correlated with C4 and C5 sugar polyols, while showing a strong negative correlation with K^+^ (Fig. 8A). However, in the bulbs of Birnförmige, Na^+^ had no significant correlation with metabolites but maintained a robust negative correlation with K^+^ concentration. In contrast, Cl^−^ in the leaves was positively correlated with ethanolamine and a trisaccharide (or a glycoside of a similar size) and was negatively correlated with at least 17 different metabolites, including ribonic acid, threonic acid, aspartic acid, malic acid, glutamic acid, glutamine, and oxoproline. The bulbs did not display any correlations with Cl^−^ treatment.

The leaves of Hytech (Fig. 8B), exhibited a strong negative correlation of Na^+^ with K^+^ and a sugar acid or lactone (< disaccharide). In the bulbs, numerous metabolites/ions, such as K^+^, 5-hydroxynorvaline (or an isomeric compound), C4 and C5 sugars, ribonic acid, and xylose, responded negatively to Na^+^. Conversely, Cl^−^ exerted a considerable influence on leaves and bulbs. In the leaves, Cl^−^ correlated positively with lysine, Ca^2+^, and a trisaccharide but correlated negatively with malic acid, ribonic acid, glyceric acid, fumaric acid, citric acid, and succinate. In the bulbs, Cl^−^ negatively affected metabolites, including dehydroascorbic acid, as well as some unidentified compounds and amino acid-like compounds.

## Discussion

### TCA cycle organic acids and few amino acids: early indicators of salinity exposure in onions

Organic acids participate in energy generation, carbon storage, and amino acid biosynthesis, enabling plants to manage excess cations and osmotic shifts. Their significance extends to influencing the taste and quality of fruits and vegetables, impacting organoleptic properties [30]. Echoing findings by Widodo et al. [12] and Pang et al. [31], this study also observes fluctuations, predominantly reductions, in TCA cycle organic acids—oxalic, malic, α-ketoglutaric, and fumaric—following salt exposure. The decrease in malate, vital for the TCA cycle, suggests a possible shift to gluconeogenesis and sugar accumulation, a response similar to that in wheat under drought conditions, implying a protective measure against stress [30, 32]. Additionally, the decrease of organic acids in the TCA cycle in both onion varieties indicates reduced metabolic activity, potentially signaling the onset of stress symptoms due to the inhibitory effects of salinity on energy production. Despite the absence of evident toxicity or adverse growth effects, reduced organic acid levels in onion plants under Na^+^ and Cl^−^ accumulation may signal the onset of the plants' adaptive response to saline conditions.

Post treatments, the landrace Birnförmige showed a decline in glutamine, potentially affecting flavor precursor synthesis. On the other hand, Hytech's lysine increment may reflect a stress-coping role and interplay with TCA-related metabolic routes [33]. The use of proline as a biomarker for mild or moderate abiotic stress, is brought into question by this study's findings, aligning with those of Lehr et al. [34] and Romo-Perez et al. [18], who propose that proline may not reliably indicate mild to moderate abiotic stress conditions across different plant species, including onions. However, we observed a significant decrease in the levels of glutamate, a precursor to proline, suggesting that proline synthesis could be poised to respond to these elevated ion concentrations and potentially upcoming severe abiotic stress as conditions worsen. These findings suggest complex regulatory mechanisms that plants deploy at the beginning to manage ionic stress, underscoring a distinct metabolic response of onions to saline conditions. Overall, the initial response of both onion varieties to salt stress involves changes in TCA cycle-related organic acids and ribonic acid. Furthermore, pathways linked to alanine, aspartate, and glutamate metabolism, depicted in Fig. 7, serve as early indicators of elevated salt conditions, affecting leaves and bulbs, particularly in the hybrid Hytech.

### Chloride effects predominate over sodium in onion plants

In response to salt treatment, both onion varieties exhibited significant accumulations of Na^+^ and Cl^−^ in leaves and bulbs (Fig. 2A-B). However, when examining the Na^+^/K^+^ ratio, a distinct contrast between the two varieties emerged. Birnförmige in particular showcased an ability to regulate its Na^+^/K^+^ balance more effectively than Hytech (Fig. 2D), suggesting robust physiological adaptations for ionic homeostasis. Despite increases in Na^+^ levels, its impact on metabolomic profiles was less pronounced than that of Cl^−^, indicating that Birnförmige may compartmentalize Na^+^ within its vacuoles to prevent metabolic disruption. This compartmentation likely reduces Na^+^ accumulation in the cytosol, thereby mitigating potential damage. This strategy, previously observed in early studies of the Birnförmige variety [18], suggests that some varieties effectively compartmentalize Na^+^ into the vacuoles, possibly mediated by a Na^+^/H^+^ antiporter. Given the relatively large vacuoles in onion cells, this Na^+^ detoxification mechanism could enable onions to withstand sodium stress at mild to moderate levels. This mechanism warrants further investigation for its potential role in onion salinity tolerance. Contrasting to the landrace Birnförmige, the higher accumulation of Na^+^ in the bulbs of the hybrid Hytech (Fig. 2A) was coupled with a decrease in sugars such as xylose and other sugars/polyols, aligns with established salt stress markers [35]. Furthermore, reductions in two derivatives of dehydroascorbic acid underscore their importance in the ascorbic acid – dehydroascorbic acid cycle (AsA-DHA), critical for plant growth and stress resilience [36, 37]. High AsA/DHA ratios, along with lower DHA levels, indicate an efficient defense against reactive oxygen species (ROS) during salinity stress, a widely observed response in plants [3]. Since bulbs of the landrace Birnförmige did not response to salt accumulation, this particular DHA alteration was exclusive to Hytech's bulbs (Fig. 7), signaling an active response to saline exposure and suggesting a defense mechanism at play.

To better understand the specific effects of Cl^−^ on onion metabolism under saline conditions, we conducted a correlation analysis (Fig. 8), providing insights into the intricate interactions among plant genetics, metabolism, and ionic responses. This analysis linked the observed metabolic alterations in amino and organic acids directly to Cl^−^ exposure. The significant downregulation of TCA cycle constituents and the modulation of pathways involving alanine, aspartate, and glutamate in both onion varieties align with similar findings in other species like faba beans, which displayed Cl^−^ buildup and associated metabolic adjustments such as the reduction of fumaric acid levels [38]. Noteworthy is the positive correlation between Cl^−^ levels and certain metabolites, including lysine in the hybrid Hytech and ethanolamine in the landrace Birnförmige, and specific trisaccharides or larger glycosides (Fig. 8), suggesting unique metabolic adaptations to chloride presence. In the hybrid Hytech, Cl^−^ accumulation triggers an increase in cellular lysine levels, emphasizing its critical role in managing salt and osmotic stress. As Cl^−^ concentrations rise, the cellular free lysine also increases, initiating significant metabolic and genetic responses. According to Arruda and Barreto 2020 [35], this elevation in lysine levels leads to the transcriptional upregulation of the LKR/SDH and AASADH genes. This upregulation facilitates the conversion of the intermediate D^1^-piperideine-6-carboxylate into pipecolate, a metabolite increasingly recognized for its contribution to plant stress responses under abiotic conditions. Furthermore, lysine metabolism intersects with proline synthesis pathways. Under stress, lysine is transformed into α-aminoadipic semialdehyde and subsequently into proline, known for its osmoprotective properties. Despite proline typically accumulating in response to osmotic stress, its synthesis did not significantly increase in our study, indicating that proline may not serve as a reliable indicator of mild to moderate salinity in onion plants.

Conversely, in Birnförmige, a marked increase in ethanolamine—crucial for choline and subsequent glycine betaine synthesis (an important osmoprotectant) [39]—might explain the less pronounced response of Birnförmige to Cl^−^ treatments compared to Hytech. Ethanolamine also plays a role in synthesizing membrane lipids, essential for preserving cellular integrity under conditions like drought and salt stress. This ability is vital for cellular function and survival under extreme environmental conditions, allowing Birnförmige to better withstand Cl^−^ exposure.

These findings underscore the dominant role of Cl^−^ over Na^+^ in the metabolic response of onions to salinity, mirroring patterns observed in other crops like *Vicia faba* L. [15, 38], and confirming dominance of Cl^−^ over Na^+^ in onions' metabolic response to saline conditions. While both onion varieties respond to stress, Birnförmige exhibits a more pronounced reaction than Hytech, highlighting its greater enzymatic efficiency in utilizing ethanolamine for stress mitigation. This capability allows Birnförmige to more effectively manage Cl^−^ stress, maintaining enhanced cellular stability and functionality compared to Hytech.

## Conclusion

This study provides significant insights into the distinct initial metabolic responses of onions to Na^+^ and Cl^−^ accumulation during the early stages of exposure to saline conditions, with Cl- playing a critical role in driving early stress-related changes. Our findings reveal that Cl^−^, rather than Na^+^, is more influential during the initial phases of salinity in altering metabolic pathways, particularly those involving organic acids in the TCA cycle. These discoveries advance our understanding of ion-specific salinity stress in onions and suggest that Cl^−^ should be given greater attention when evaluating plant responses to saline environments. The ability of certain varieties, such as the landrace ‘Birnförmige,’ to regulate its Na^+^/K^+^ balance more effectively than the hybrid ‘Hytech’ highlights potential opportunities for breeding programs aimed at improving salinity tolerance in onions, depending on the variety. Future research should explore the long-term physiological and agronomic consequences of Cl^−^ accumulation in onion plants, as well as extend this ion-specific approach to other crops, particularly vegetables sensitive to salinity. Expanding these findings to different environmental conditions and onion varieties could help refine strategies to enhance crop resilience in saline-affected agricultural systems.

## Supplementary Information

Supplementary Material 1.

## Data Availability

Data are provided within the manuscript and its Supplementary Information files. Datasets and the associated analytical codes are available upon request from the corresponding author, M.L. Romo-Pérez, who can be contacted at m.romoperez@uni-hohenheim.de.
