# Supplementary material for: The tale of two Ions Na+ and Cl−: unraveling onion plant responses to varying salt treatments
Source: BMC Plant Biol. 2024 Oct 29;24:1022. doi: 10.1186/s12870-024-05719-9 (PMC11520526; doi:10.1186/s12870-024-05719-9)
Supplement: Supplementary file 1 — Supplementary Material 1. [file 12870_2024_5719_MOESM1_ESM.docx]

**The Tale of Two Ions Na^+^ and Cl^-^: Unraveling Onion Plant Responses to Varying Salt Treatments**

**M. L. Romo-Pérez^1^**, C. H. Weinert^2^, B. Egert^2^, S. E. Kulling^2^, C. Zörb^1^

^1^ University of Hohenheim, Institute of Crop Science, Quality of Plant Products 340e, Schloss Westflügel, 70599 Stuttgart, Germany. ^2^ Max Rubner-Institut, Department of Safety and Quality of Fruit and Vegetables, Haid-und-Neu-Straße 9, 76131 Karlsruhe, Germany

**Corresponding authors**

M.L. Romo-Pérez: [m.romoperez@uni-hohenheim.de](mailto:m.romoperez@uni-hohenheim.de)

C. Zörb: [Christian.zoerb@uni-hohenheim.de](mailto:Christian.zoerb@uni-hohenheim.de)

***Keywords:*** *Salinity, Allium Cepa L., Sodium (Na^+^), Chloride (Cl^-^), Organic Acids, Tricarboxylic Acid Cycle (TCA), Metabolomics*

Table S1 Basic properties of soil mixture at the transplanting time before general fertilization. EC, electrical conductivity

| *pH-value* | *NH4-N*  *mg/kg:* | *NO3-N*  *mg/kg:* | *S_min_*  *mg/kg:* | *K*  *mg/kg:* | *P*  *mg/kg* | *EC*  *mS cm^-1^* |
| --- | --- | --- | --- | --- | --- | --- |
| 7.02 | 4.0 | 4.1 | 13.1 | 5.9 | 7.4 | 0.23 |


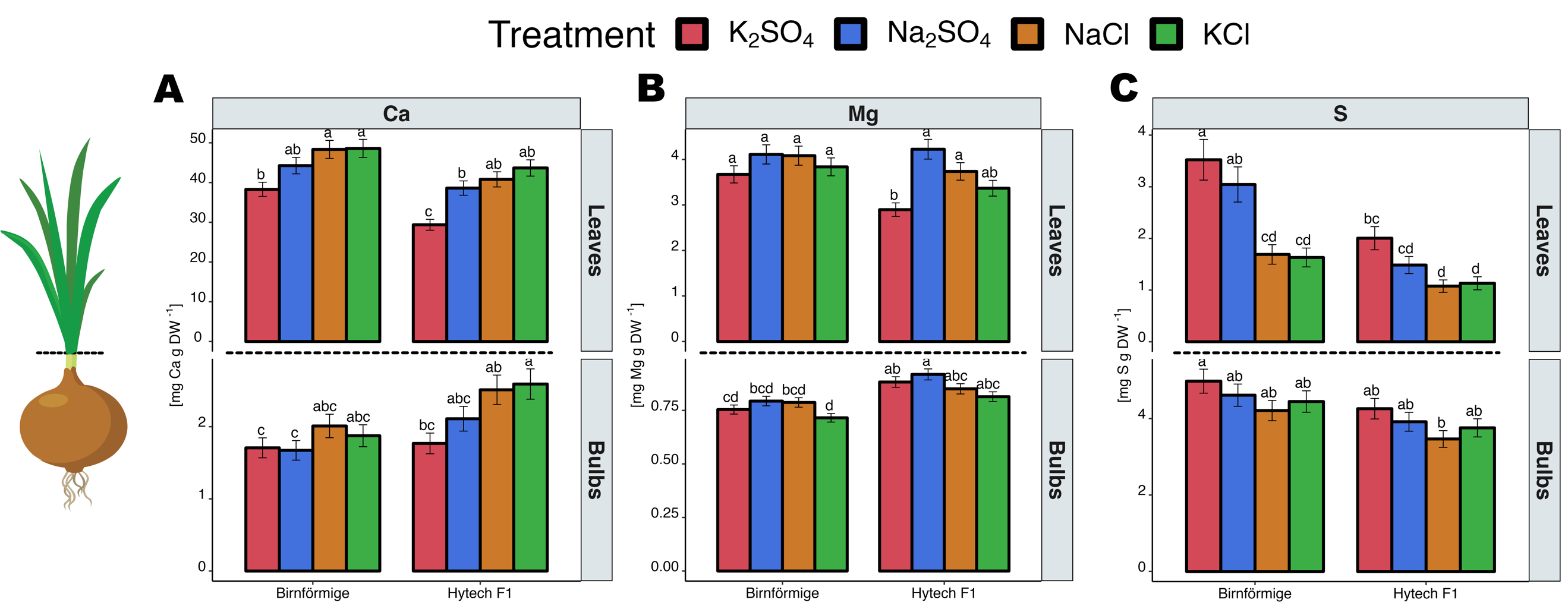


Figure S1 Concentration of Ca^2+^, M ^2+^ and total sulfur in onion bulbs and leaves


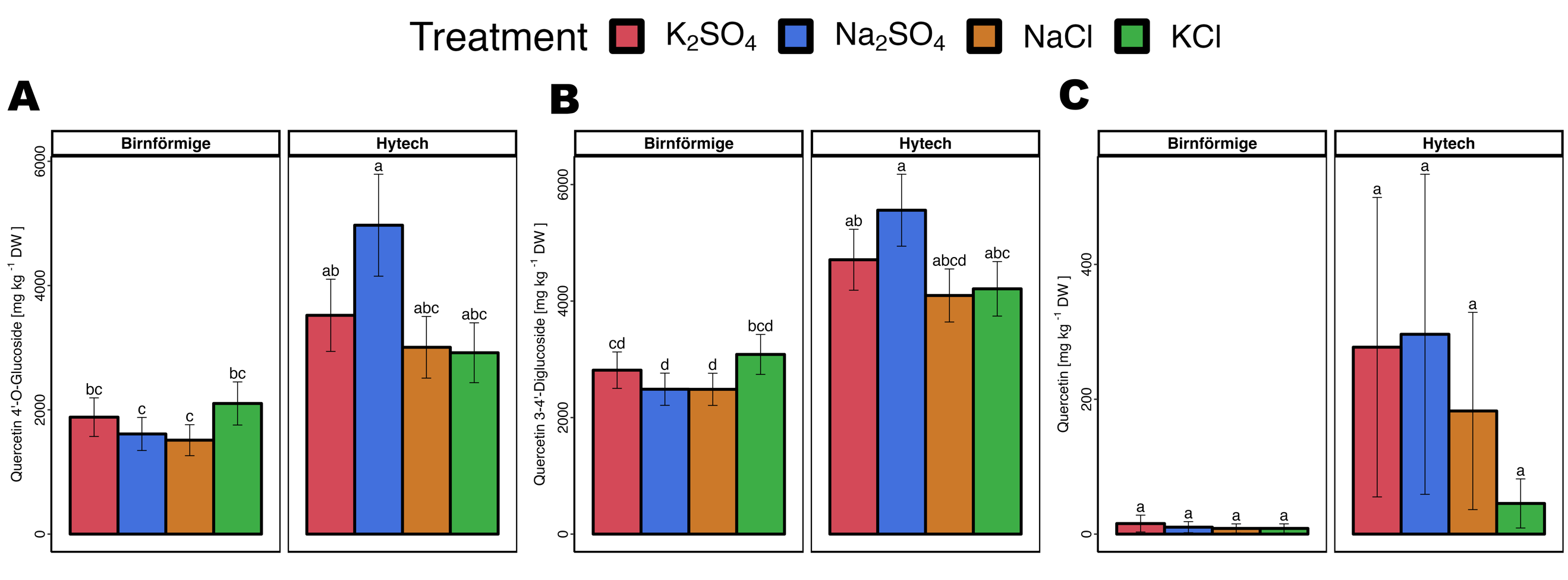


Figure S2 Quercetin concentration in onion bulbs. A: Quercetin-4’-O-Glucoside, B: Quercetine 3-4-Diglucoside, and C: Quercetin.
